# Planning to be incremental: Scene descriptions reveal meaningful clustering in language production

**DOI:** 10.1016/j.cognition.2025.106330

**Published:** 2025-09-22

**Authors:** Karina Tachihara, Madison Barker, Beverly Cotter, Taylor Hayes, John Henderson, Adrian Zhou, Fernanda Ferreira

**Affiliations:** a Department of Linguistics, University of Illinois, Urbana-Champaign 707 S. Mathews Ave., Urbana, IL 61801, United States; b Department of Psychology, University of Illinois, Urbana-Champaign 603 East Daniel St., Champaign, IL 61820, United States; c Department of Psychology, University of California, Davis 135 Young Hall, 1 Shields Ave., Davis, CA 95616, United States; d Center for Mind and Brain, University of California, Davis 267 Cousteau Pl., Davis, CA 95618, United States

**Keywords:** Linearization, Multiutterance production, Clustering, Scene description

## Abstract

How do speakers plan complex descriptions and then execute those plans? In this work, we attempt to answer this question by asking subjects to describe complex visual scenes. We posit that speakers begin planning by organizing the scene into meaningful clusters or groupings of objects. Speakers describe the scene cluster by cluster, allowing for some planning time between each cluster. To test these ideas, in a preregistered study 30 participants described 30 indoor and outdoor scenes while their speech was recorded. Physical distance was calculated by identifying the centroid point of each object and then computing the Euclidean distance between centroid points for every object pair. Semantic distance was calculated using ConceptNet Numberbatch to obtain the semantic similarity between object labels. A clustering algorithm was then applied to establish the appropriate number of clusters per scene and to assign objects to each cluster. We observed that, consistent with our hypothesis, objects separated by shorter physical distances and objects that are semantically more similar were discussed in closer temporal proximity in the verbal descriptions. In addition, word productions that involved jumping from one cluster to another took longer to initiate than those associated with the same cluster. We conclude that speakers address the linearization problem by establishing clusters of objects and using them to facilitate incremental planning. This approach treats multiutterance language production as a type of foraging behavior, where people balance exploration and exploitation.

## 1. Introduction

Language production is a complex task involving multiple steps from thought formation to motor coordination. Adding to this complexity is the fact that we often want to convey multiple ideas that are connected to one another, rather than just a single concept. To successfully convey multiple ideas, it is necessary to order them so that they can be produced in a sensible sequence. Linearization, or the ordering of ideas, is a core problem in language production. For instance, if we want to talk about multiple objects, we need to decide which object will be mentioned first, second, etc^1^. [^1^In this paper, we concentrate on production in the form of speaking, however, we believe that, in some cases, similar principles may apply to writing or signing.] The linearization problem was first discussed in Levelt (1981) and expanded on in his book, *Speaking* (1989).

Linearization occurs at multiple levels of the production process, from conception to articulation. Levelt (1989) had proposed a macroplanning stage and a microplanning stage, where the former is about the choice of communicative goals and subgoals while the latter is about the choice of the structure of the preverbal information (see also Butterworth, 1980). Besides the type of information being linearized, the stages differ in the scope of production. Much of the literature on linearization has dealt with the microplanning stage, concentrating on the ordering of words in phrases and single sentences. In this paper, we tackle the problem of macroplanning, by analyzing multiutterance production evoked by a rich stimulus and the specific linearization issues that arise with it.

Linearization is not only deciding what comes earlier or later but also when to make those decisions during speech planning. One possibility is that planning occurs fully before production. To get an intuition for this, consider the most extreme version of pre-planning: The speaker determines the entire sequence of what they intend to say and how they will say it before starting to speak. This would require that all ideas, words, and sounds be represented and ordered upfront, and that the speaker would hold this full plan in memory while articulating each linguistic piece sequentially. This strategy has the advantage of eliminating the need to plan amid production, allowing, theoretically, for a fluid production with no interruption (Brown-Schmidt & Tanenhaus, 2006) or an “ideal delivery” (Clark & Clark, 1977). However, the approach would also come with substantial costs: a heavy cognitive load at speech onset, significant demands on working memory, and delayed production at initiation. Even proponents of the message-first view of language production, such as Wundt (1900), did not propose such comprehensive and detailed linguistic prespecification; their focus was on the conceptual structure of the utterance and not its full linguistic realization.

An alternative theory is that deciding what to talk about and how to talk about it occurs as production unfolds (Bock & Levelt, 1994; Kempen & Hoenkamp, 1987). Consider now a radical incremental view. On this view, planning and speaking proceed in tight alternation: While producing one unit, the speaker plans the next. This allows for earlier speech initiation but may require a great deal of on-the-fly message formulation and reformulation at each step, potentially slowing or disrupting fluency.

In this paper, we adopt a view of incrementality that differs from the radical version by incorporating periods of planning with periods of articulation. We also aim to better identify how speakers might manage being incremental. We hypothesize that multiutterance production involves incremental planning through clustering. In this view, a speaker interleaves planning and articulation by clustering parts of their messages into more manageable, bite-sized chunks. We believe that by using clusters to create breaks in the stream of processing events required for production, the speaker can leverage the advantages offered by both planning and incrementality. Namely, speakers would be able to start speaking after only some planning, use cluster boundaries as a designated time to plan, and burden working memory less overall. We believe incremental planning through clustering is especially useful for multiutterance productions that include many ideas. This proposal differs from the radical planning and radical incrementality views described above but is harmonious to views of production that include some planning and some incrementality. The novel contribution of this strategy comes from the incorporation of clustering as a way to balance planning and incrementality. Because few studies have investigated macroplanning and production at the multiutterance level, we relied on the related production literature to inform our hypothesis. In this paper, we use the term, linearization, broadly to encompass all levels. While we acknowledge that macroplanning and microplanning could be distinct processes, we were inspired by past studies on linearization when theorizing how multiutterance descriptions might be organized. Therefore, we briefly review support for incrementality as well as planning in the microplanning production literature before returning to the advantages of clustering. We then present our task and its benefits when testing the idea of incremental planning through clustering.

### 1.1. Incremental production

Support for incrementality comes from production experiments which analyze initiation times for articulation. Wheeldon and Lahiri (1997), for example, found that initiation times were affected by the phonological activation of the earlier part of the sentence, but not the later parts. In an experiment where participants were given the nouns and asked to produce a clause, Ferreira (1996) found that production of verbs with flexible order was quicker because it allowed for the more accessible word to be produced sooner. Past work looking at ordering preferences in the production of nouns in phrases and sentences has found that words that are more animate, previously referenced, and generally more cognitively accessible are likely to be mentioned first (Christianson & Ferreira, 2005; Griffin & Bock, 2000; MacDonald, 2013; Morgan & Levy, 2016; Tachihara & Goldberg, 2020). The placement of more cognitively accessible concepts, or concepts that can be activated in the mind relatively easily, earlier in the utterance is consistent with an incremental system that chooses a noun as it produces the utterance.

### 1.2. Planning in production

On the other hand, there is also evidence for some planning before production begins. In Ferreira and Swets (2002), participants produced computations of arithmetic sums (21 + 4), such as *the answer is 25*, *25 is the answer,* or *25*. The difficulty of the problem did not change utterance duration, suggesting that participants did not begin their utterance before they had decided on the answer. When asked to speak as quickly as possible, however, utterance duration showed an effect of problem difficulty, suggesting that participants were calculating and deciding on the answer while they spoke. In both tasks, difficult problems led to later initiation times. Interestingly, the accuracy of the arithmetic calculation did not drop when initiation times were cut short due to the demands of the task, suggesting that participants take extra time after they decide on the answer to start speaking. From these results, the authors ruled out a radical incremental view with little to no planning, and instead suggest a strategic incremental view, where some planning occurs before speech initiation when circumstances allow.

Studies of syntactic priming can also be considered as support for some level of planning before production. In Bock (1986), participants were primed with either an active or a passive frame (*One of the fans punched the referee*. vs. *The referee was punched by one of the fans.*). When describing a new image, participants were likely to produce the primed framing (*The lightning is striking the church.* vs. *The church is being struck by lightning*). Since activation of the active or the passive frame involves deciding the order of the two nouns, rather than relying on the accessibility of one, it can be considered as a planned utterance.

### 1.3. Problem of multiutterance production

**Fig. 1.**
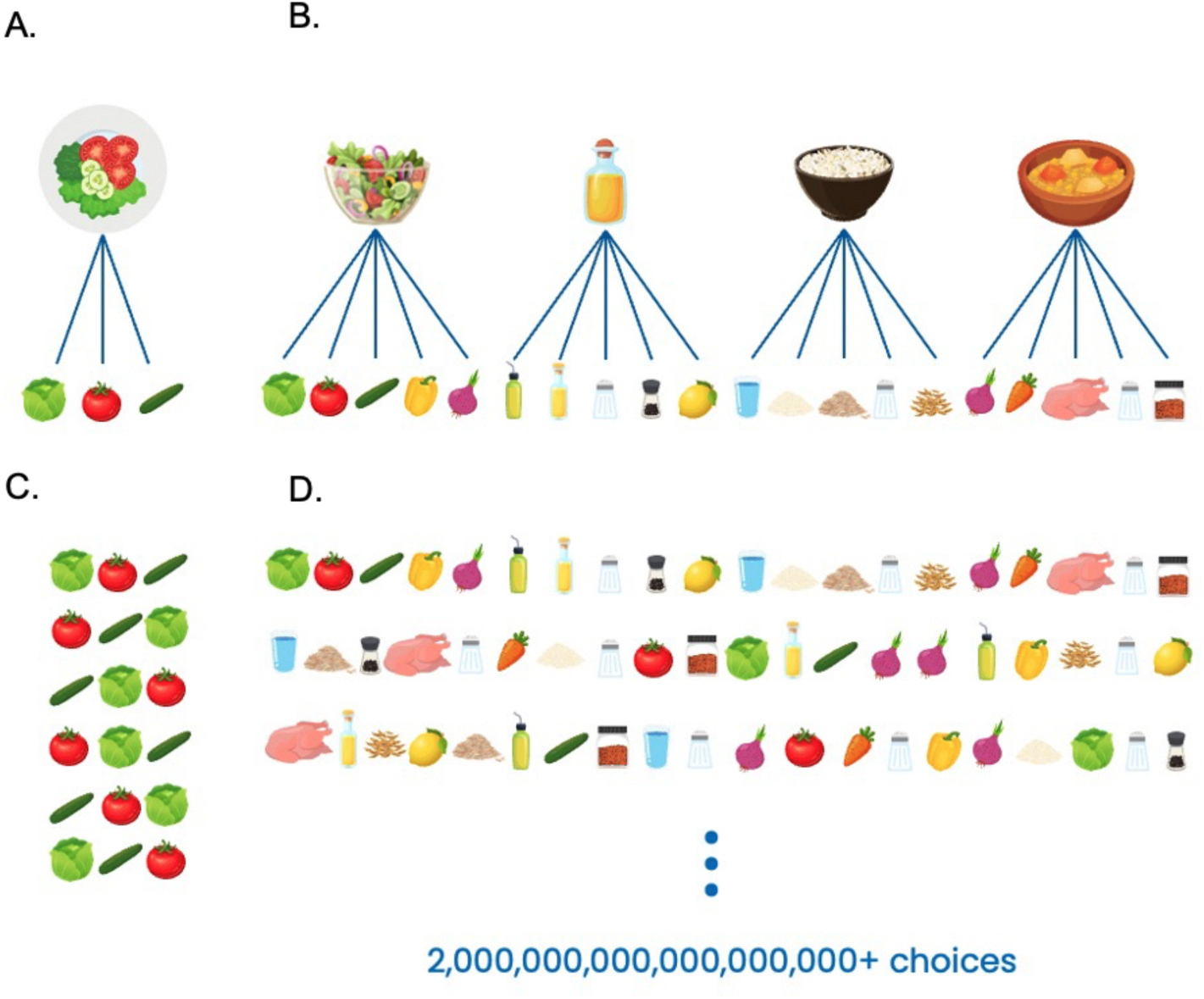
Example of a simple vs. complex linearization problem in cooking.

Past studies of linearization have focused on the order of a couple of nouns in phrases and sentences, where there were few choices, such as using the active or passive alternation (Griffin & Bock, 2000) or binomial expressions (Morgan & Levy, 2016; Tachihara & Goldberg, 2020). When there are two choices in particular, choosing the first element automatically decides the entire order because the other element must come second. However, whether the speaker plans or not may differ on the type and length of production. In multiutterance production, there are many ideas to be conveyed that are interconnected, with many choices for how they can be organized. In such a case, planning may play a larger role. For example, imagine preparing a salad versus a meal (Fig. 1). If the ingredients are just lettuce, tomatoes, and cucumbers, it may not matter the order in which you prepare them (Fig. 1A & C). You can start with whichever vegetable is closest to you. Now imagine you are making a salad, a salad dressing, a chicken curry, and rice (1B). If you start with whatever is available to you first and then move on to the next ingredient, you may make several mistakes. For example, if you start by cutting the raw chicken, you will have to wash the cutting board and knife before cutting the raw vegetables so that the vegetables are not contaminated. However, if you plan the order of preparation strategically, you can cut the vegetables for the salad first and then cut the raw chicken without having to wash the cutting board and the knife in between, saving you some time and effort. You will also want to make sure that you don’t move from one dish to another prematurely. If you forget whether or not you have already added salt to the salad dressing, you may end with a salad that is too salty and a chicken curry that is too bland. Therefore, the best strategy may be to plan the order in which you will cook your dishes while allowing for some flexibility in the order of ingredients within a dish.

### 1.4. Clustering

Hierarchical organization is ubiquitous in language and other cognitive domains (Lashley, 1951). Research on planning for complex tasks emphasizes the spontaneous creation of a hierarchical structure across domains. Hierarchical organization has been observed in spatial representation during navigation (Wiener & Mallot, 2003), event segmentation (Kurby & Zacks, 2008), and action sequences (Cohen, 2000). Recent developments of computational models capture how hierarchical structures are learned and how they are later used for planning (Correa et al., 2023; Schapiro et al., 2013; Tomov et al., 2020). Across different experimental paradigms, researchers have found clusters containing several components of a sequence to complete the task^2^. [^2^ A hierarchical organization would also imply layers of sub clusters, which itself may contain sub clusters. Although this may also be the case in language production, in this paper, we concentrate on one single layer of clusters.]

A plan containing clusters has several advantages over “flat” or a purely sequential plan. First, humans have working memory limitations that may hinder or prevent remembering each step of a sequence. We have long known that the number of items that one can recall increases if items can be clustered together or chunked (Miller, 1956). Clusters that are related to familiar concepts, or in other ways connected to long-term memory, have a stronger effect, but even clusters that are created on the fly and live in working memory lead to improvement in the number of items that can be recalled (Thalmann et al., 2019). One of the tasks in linearization is bookkeeping, or the need for the speaker to store what they have talked about and what they have yet to talk about in order to produce a coherent set of utterances that do not repeat information unnecessarily (Levelt, 1989). If clustering can help memory recall, it could also be helpful in bookkeeping during production by improving memory for the contents of production.

Second, planning using clusters reduces the amount of planning time by reducing the number of components that must be involved at a given time. In the cooking example we illustrated, one planning strategy may be to cluster the ingredients for the meal into dishes. Instead of having to linearize the order of 20 ingredients, you need only to plan the order of the four dishes. Once you are starting to cook each dish, you can then plan how to incorporate each of the five ingredients for that dish. Mathematically, this reduces your choices from a permutation of 20 ingredients, which is over 2 quintillion unique sequences (Fig. 1D) to a permutation of four dishes plus permutations of five ingredients repeated for each of the four dishes, which is significantly lower with a total of 504 choices (24 + 120 × 4).

Lastly, planning with clusters allows planning to be interleaved with production. You do not need to wait until you sequence all 20 ingredients before starting to cook. You can start cooking the first dish as soon as the order of the dishes has been planned, before deciding the order of ingredients for the rest of the dishes. Planning with clusters, then, may facilitate incremental production, and specifically the interleaving of formulation and articulation processes. Levelt (1989) discusses the idea that it is not necessary for macroplanning to be complete before microplanning can begin. However, the timing of when the speaker should switch from one to the other is underspecified. Past work on speech fluency supports the idea of interleaving stages. Roberts and Kirsner (2000) found that participants’ speech followed fluency and disfluency cycles, and that these cycles were marked with a topic shift (see also Goldman-Eisler, 1968; Greene & Cappella, 1986; Merlo & Barbosa, 2010). These topic shifts, coded using subjective ratings in Roberts and Kirsner (2000), are similar to the idea of jumping clusters, which we operationalize in this paper using a data-driven approach.

Spontaneous use of clusters has been observed in another complex task: foraging. In a verbal fluency task (Hills et al., 2015), participants were asked to name as many items as possible from a given category (e.g., animals). They found that participants followed a pattern of local exploitation within related items (ex: cow, horse, pigs) and global exploration of new clusters (ex: lion, zebra, cheetah). Both internal foraging tasks, such as naming items from memory, and external foraging tasks, such as looking for apples in trees or information on the internet, make use of patches or clusters to balance local exploitation (finding nearby items) and global exploration (traveling far to find new items) for optimum gain (Pirolli & Card, 1999; Todd & Hills, 2020).

### 1.5. Scene descriptions

We hypothesize that the linguistic production of complex ideas requires a strategic approach where clusters are used to interleave planning with production. In the current study, we investigate linearization strategies in multiutterance production of scene descriptions. Participants were asked to freely view and describe naturalistic images without animate entities. Scene descriptions have several desirable characteristics to answer the questions at hand. First, scene description is an intuitive, open-ended task that allows many choices to be made by the participant. The participant is free to decide what to talk about, what not to talk about, what to mention earlier or later, how much to talk about each component as well as how much to plan and when to plan their utterance. Second, real-world scenes are naturally complex, where the objects within them have relationships with each other as well as the scene (Henderson & Hollingworth, 1999). This property is essential because we want to investigate how people tackle complex production tasks that consist of interdependent steps. Third, because the descriptions are related to the picture they are viewing, we are able to map their verbal responses to the objects in the image, making the mentioned concepts concrete. Lastly, visual attention has been known to affect subsequent linguistic expressions (Clarke et al., 2013; Gleitman et al., 2007; Griffin & Bock, 2000; Montag & MacDonald, 2014). Scene viewing is an established method within visual attention and has a rich literature that can inform us about the relevant features that guide people’s attention in naturalistic scenes.

One such tendency is for people to attend to semantically rich areas of a scene. Several studies using eye-tracking during free viewing have found that fixation location is better explained by semantic meaningfulness compared to visual saliency (Hayes & Henderson, 2022; Henderson & Hayes, 2018; Peacock et al., 2020). Object relationships also play a role, where areas with objects which were more semantically related to the scene label and other objects were more likely to be fixated (Hayes & Henderson, 2021). Color, on the other hand, unless specifically instructed to do so, had little impact on eye movements (Buswell, 1935).

Similar effects were found when participants described scenes or prepared to describe scenes. Semantic meaningfulness of an area was a significant predictor of where people looked when controlling for center proximity, a factor that has been separately shown to influence overt attention (Ferreira & Rehrig, 2019; Henderson et al., 2018; Rehrig et al., 2020). Interestingly, the initiation of the verbal descriptions showed an average latency of 1700 ms (SD = 700 ms) after scene onset and before speech onset (Barker et al., 2023). This latency is notably longer than what would be expected from the time it would take for retrieval of the first word based on a visual cue, which is approximately 812 ms (Costa et al., 2009). It is also significantly longer than the time it takes to capture the scene gist, which takes less than 100 ms (Oliva, 2005), or make the first fixation, which is approximately 250–300 ms (Henderson et al., 2018). In an EEG study of scene viewing, a link between ERPs and the semantic distribution of the scene emerged as early as 87 ms (Kiat et al., 2022). Thus, the latency in speech initiation likely reflects the time in which participants are planning their speech.

Work on verbal descriptions has been more limited. Shanon (1984) asked participants to describe familiar rooms and annotated their descriptions into parts and subparts, showing that descriptions progressed from general to specific. Barker et al. (2023) analyzed participants’ descriptions of scenes and found that object affordances predicted the order of mention. Specifically, they collected judgment data on the object’s interactability, or the degree to which subjects believed a human would interact with that object, and they reported that objects that were rated as higher in interactability were mentioned earlier in the utterance. Objects which were closer to the center were also mentioned earlier, consistent with center bias in visual attention. They also found an effect of object size, where larger objects were mentioned earlier. This is again consistent with findings from scene viewing which suggest that large objects representative of the space guide people’s attention (Võ et al., 2019). Clarke et al., 2013 investigated object naming from naturalistic scenes and found that both visual attention to objects and linguistic features, such as animacy and semantic proximity, influenced speakers’ decisions to name an object or not. They suggest that visual features and linguistic features interact and constrain each other rather than work independently during this cross-modal cognitive task (also see Coco & Keller, 2012).

In the current paper, we focused on how we organize our speech during multiutterance descriptions of scenes without animate entities and examined the influence of relationships among objects. Given prior work on scene viewing and scene descriptions, we examined object relationships across four features: physical location, semantic relations, center proximity, and size. We hypothesized that participants’ verbal descriptions would show evidence of incremental planning through clustering. Specifically, we predicted that the timing of object mention will reflect the clustering of objects in the scene. We quantified clustering in two ways. First, we looked at whether objects that are close together based on relationships extracted from the image were mentioned close together in the verbal description. Second, we used the object relationships to create predefined clusters in each of the four features and examined whether the time it took between mentioning one object to the subsequent object was affected by cluster boundaries. We further examined boundary crossings to see if there were linguistic signs of planning by looking at the length of pauses and instances of filler words.

## 2. Methods

Data were collected as part of a larger set of studies investigating scene description and visual attention. The following sections were duplicated from Barker, Rehrig and Ferreira (2023) and Hayes and Henderson (2021): Participants, Stimuli, Procedure, and portions of Object Relations. In this paper, we report results from a new dataset, but focus only on the verbal descriptions collected; eye tracking data will be presented in a subsequent article. Data collection took place in the spring of 2022 during the COVID-19 pandemic. Researchers and participants followed safety protocols, such as wearing a mask.

### 2.1. Participants

Thirty-three undergraduate students with normal or corrected-to-normal vision were recruited at University of California, Davis. Two were excluded because they could not be accurately eye tracked. One was excluded because they had taken part in the pilot stage of the same experiment. We report results from 30 subjects. Participants were naive to the purpose of the experiment, provided informed consent prior to participation, and received course credit for their participation.

### 2.2. Stimuli

Thirty real-world images from indoor and outdoor scenes were used as stimuli. Scenes did not include animate entities, except for one scene that contained a cat.

### 2.3. Procedure

The experiment began with a five-point calibration procedure for the eye-tracker. Subjects were provided the following instructions: “In this experiment, you will see a series of scenes. You will have 30 seconds to describe the scene out loud.” Prior to the experimental portion, subjects completed three practice trials. At the start of each trial, subjects fixated on the center fixation in a five-point fixation grid until the experimenter keyed forward to begin the trial. The scene was presented for 30 s while eye movements and speech were recorded. Subjects were presented scenes in a pseudo-random order such that scenes that depicted the same category did not occur consecutively (e.g., two different kitchen scenes in sequence). After the 30 s response window ended, subjects initiated the next trial by pressing any button. Because we were interested in how participants organized their description of the scenes, only the verbal data were analyzed for this study.

### 2.4. Analysis

#### 2.4.1. Scene segmentation

**Fig. 2.**
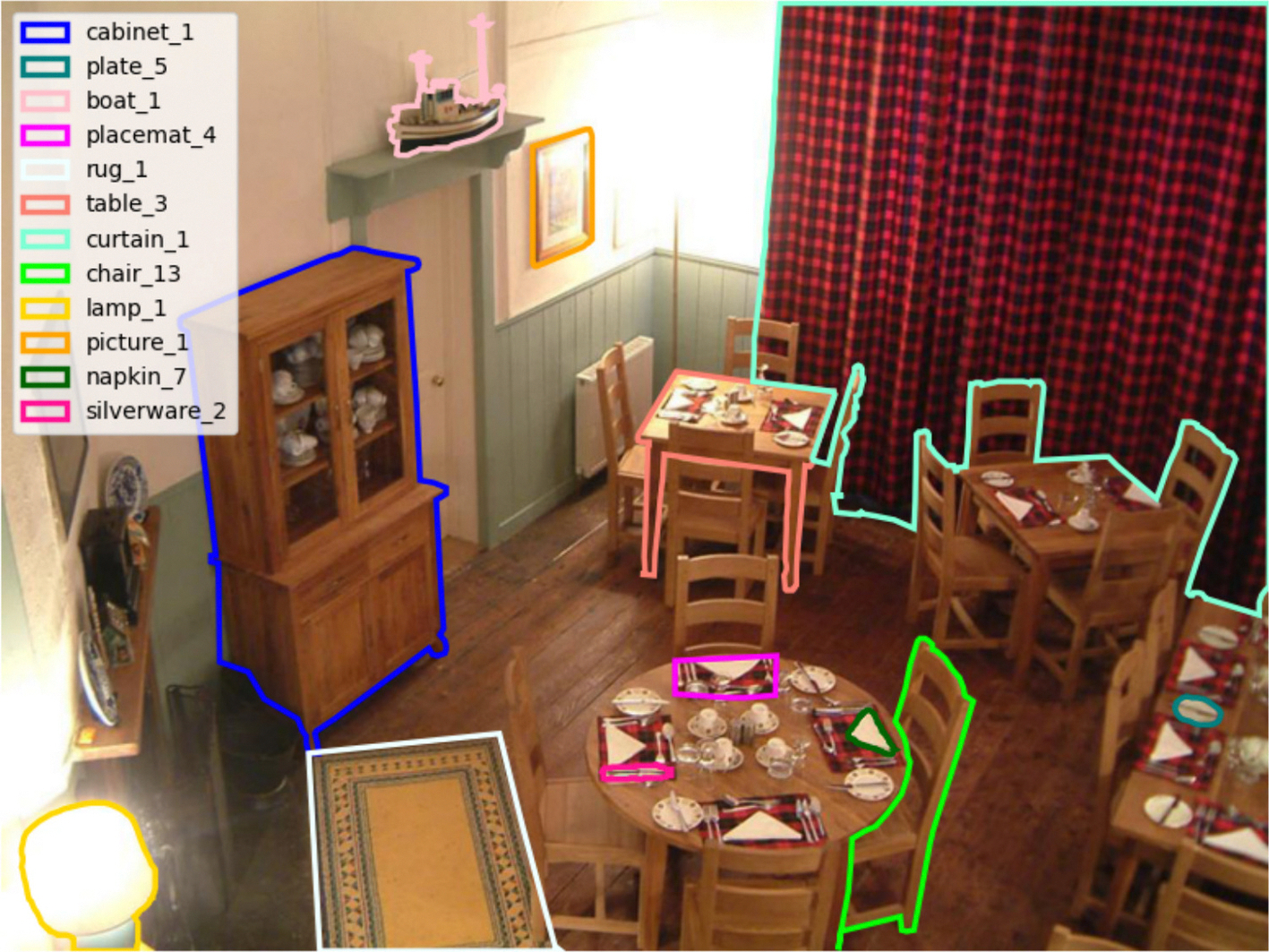
Example scene with object segmentation. For the purpose of visualization, we chose not to show all objects since it makes it difficult to see the object segmentations. Instead, we chose 12 objects in this image to show its segmentations and labels. Each object boundary is shown with a different line color. The matching of the color and the label can be found on the top left corner.

Each scene was segmented into objects with unique labels. The list of object labels for a given scene was defined prior to the analysis of the verbal data. We used AnyLabeling (Nguyen, 2023) to identify the object boundary for each object and to give it a label. AnyLabeling has an automatic object segmentation tool, where object boundaries are assigned given the image using one of many models. We used the Segment Anything Model (ViT H Quant; Kirillov et al., 2023). Most objects were identified using this automatic object segmentation tool. If the model was unable to segment the object appropriately, we manually created the object boundaries by specifying points around the object. Fig. 2 shows an example of a scene with a subset of segmented objects.

Each scene was segmented exhaustively, including ceilings and floors. Our goal was to minimize gaps as much as possible so that every space in the scene belonged to at least one object. We calculated the number of pixels that belonged to at least one object and the number of pixels that did not belong to any object and confirmed that for every image at least 95 % of pixels belonged to an object. Wherever possible, overlap between object segmentations was avoided. Exceptions included parts of objects, transparent objects (ex: cups inside a glass cupboard such as in Fig. 2) and objects that were too small to be segmented separately, which accounted for 18.7 % of pixels. (for additional guidelines on segmentation, refer to Supplemental Information 1).

#### 2.4.2. Object relations

Because we are interested in how the relationships between objects in a scene affect descriptions, we calculated the relationship between every object in a scene to every other object in a scene. We calculated relationships between pairs of objects for each of the four features of interest: physical distance, semantic distance, center proximity difference, and size difference.

For physical distance, we first identified the centroid point for each object using the boundaries defined during segmentation. We then calculated the Euclidean distance between the centroid points for each object pair.

For semantic distance, we used the method previously reported in Hayes and Henderson (2021). We took the inverse of the semantic similarity score between two object labels (1 minus semantic similarity score). ConceptNet Numberbatch (Version 17.06; Speer et al., 2017) was used to estimate the semantic similarity between object labels as vectors in a high-dimensional space. ConceptNet Numberbatch uses an ensemble approach combining the semantic vectors from Word 2vec (Mikolov et al., 2013) and GloVe (Version 1.2; Pennington et al., 2014)—which learn how words are associated with each other from large text corpora (i.e., Google News, 100 billion words; Common Crawl, 840 billion words)—with ConceptNet, a knowledge graph that draws on expert-created resources (WordNet, Fellbaum, 1998; Open Mind Common Sense, Singh et al., 2002; OpenCyc, Lenat & Guha, 1989) and crowdsourced knowledge (Auer et al., 2007; Kuo et al., 2009; von Ahn et al., 2006). The benefit of the ConceptNet Numberbatch ensemble approach is that it produces high-quality semantic representations that are better than any single component of the ensemble (e.g., Word2vec) on a number of important semantic benchmarks, such as SAT analogies (Speer et al., 2017). We then used the generated ConceptNet Numberbatch semantic vectors to compute how semantically related the objects in each scene were to one another. Specifically, we computed the similarity between each pair of object-label vectors using cosine similarity (i.e., the normalized dot product of the two-word vectors) (Damiano et al., 2024; Hayes & Henderson, 2021). Cosine similarity tends to be highest for category members that appear in similar contexts and that share many features such as “chair” and “couch.” Therefore, items like “chair” and “couch” would have a short semantic distance compared to items like “chair” and “boat”.

For center proximity differences, we first computed the center proximity of each object by taking the Euclidean distance between the centroid point and the center of the image (Barker et al., 2023; Hayes & Henderson, 2020). We then calculated the absolute value of the difference in center proximity between each object pair.

Finally, for size differences, we first computed the area within the boundaries defined during segmentation for each object. We then calculated the absolute value of the difference in the sizes for each object pair.

#### 2.4.3. Clustering

**Fig. 3.**
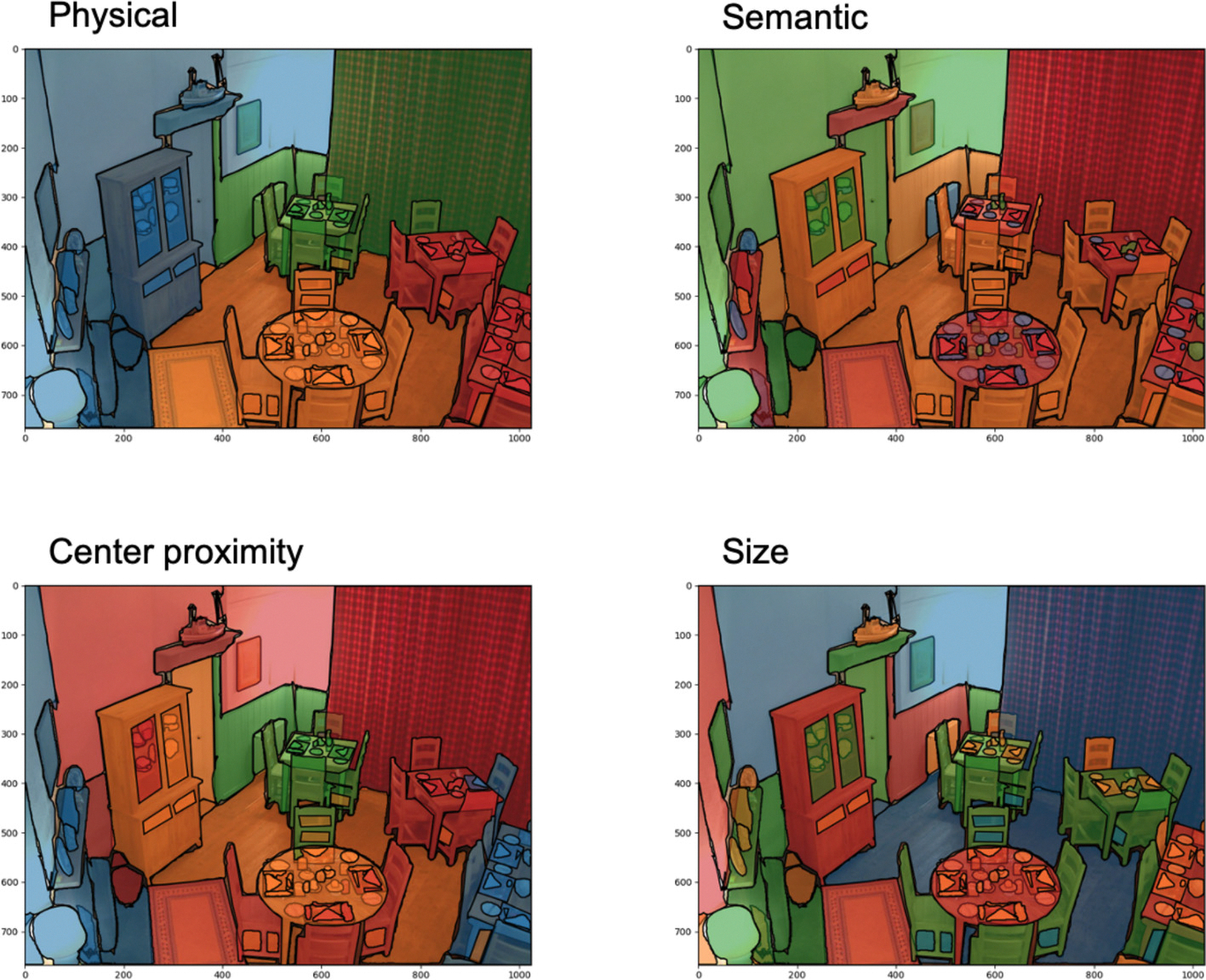
Example scene and object clustering for each feature: physical cluster (top left), semantic cluster (top right), center proximity cluster (bottom left), and size cluster (bottom right). Objects with the same coloring belong to the same cluster.

We took a data-driven approach to object clustering. In order to avoid circularity, clusters were identified using properties of the objects from the image, rather than participants’ descriptions. We then analyzed whether the descriptions were influenced by these preidentified clusters. Objects were grouped using the pairwise distances and differences calculated in Section 2 on object relations. Separate sets of clusters were created for each of the features: physical distance, semantic distance, size difference, and center proximity difference (Fig. 3). We used the k-medoid clustering algorithm within the scikit-learn-extra package to cluster the objects (Pedregosa et al., 2011). We found the optimal number of clusters by calculating the silhouette score for every cluster size between two and the number of objects in a scene minus one and then choosing the cluster size with the lowest silhouette score. Given this approach, each feature had a different optimal cluster number for a given scene. In order to compare across clusters, we took the lowest optimal cluster number across features and set that to be the cluster size for a given scene across all features. Because the clustering algorithm we used is based on a hierarchical model, cluster numbers that are smaller than the optimal are still meaningful (i.e., if five clusters is a meaningful number of clusters, four clusters are also meaningful whereas six clusters might not be meaningful). Fig. 3 shows the object clustering for each feature for the same scene.

#### 2.4.4. Verbal data coding

The recordings were transcribed using a speech-to-text algorithm, Whisper (OpenAI), and then manually corrected. We set the parameter of the algorithm to be sensitive to filler words, so that the resulting transcript contained instances of “uh”, “um”, and “umm.” The onset and offset of each word in the descriptions were identified using the Penn Phonetics Forced Aligner (p2fa) (Yuan & Liberman, 2008). Using the timestamped transcription, we identified the objects mentioned. For each description of a scene by a participant, an undergraduate research assistant read the transcript, identified the word referring to an object or objects, and identified the object(s) in the scene by looking at a scene alongside the transcription. The words that were identified included both object names and pronouns with identifiable referents (ex: “It” in “There’s a big wooden table. It has a plant on top.”). Objects that were mentioned multiple times made up 1.5 % of objects. In 50 % of descriptions, participants described the scene in general (ex: “This is a kitchen.”), often at the beginning. This did not count as a mentioned object. Objects in locative prepositions (ex: “table” in “There is a book on the table.”) were included as mentioned objects. These accounted for roughly 3 % of mentioned objects. For additional guidelines on verbal data coding, refer to Supplemental Information 2.

#### 2.4.5. Verbal relations

To look at how objects were organized in a description, we analyzed the pairwise relationships between all the objects mentioned within a description to every other object mentioned in the same description. For each relationship, we identified the physical distance, semantic distance, size difference, and center proximity difference, calculated in the same way as described in Section 2 on object relations. We also categorized each relationship as a “jump” if the two objects did not belong to the same cluster and as not a jump if they were in the same cluster. Each relationship was then given a weight according to how many objects were identified for each word. This was done so that each mention was weighted equally important and informative in the model analysis whether it referred to one object or many objects. The weight was calculated as (1/number of objects referred to by the first word * 1/number of objects referred to by the second word). For example, if a participant talked about a table and some chairs, where chairs referred to 14 different instances of chairs like in Fig. 3, the relationship between the table and each chair would each receive a weight of 0.07.

## 3. Results

### 3.1. Preregistration & open science

We preregistered the study’s hypotheses, dependent measures, and primary statistical analyses on AsPredicted.org before data analysis (https://aspredicted.org/JB6_M5J). The data were collected as part of a previous study but had not been processed or analyzed before preregistration. We based our preregistration on a previously collected pilot data set. Deviations from the preregistration are explicitly stated as exploratory and explained in the text with additional references to supplemental information. We considered the preregistration to be particularly useful given the novelty of our hypotheses and analyses; our aim was to reduce the appearance of only reporting the significant analyses after exploration. Data and analysis code are available on Open Science Framework (https://osf.io/nymbh/?view_only=5df98bff1a4045a688685f6b1065db54).

### 3.2. Distance between objects

**Fig. 4.**
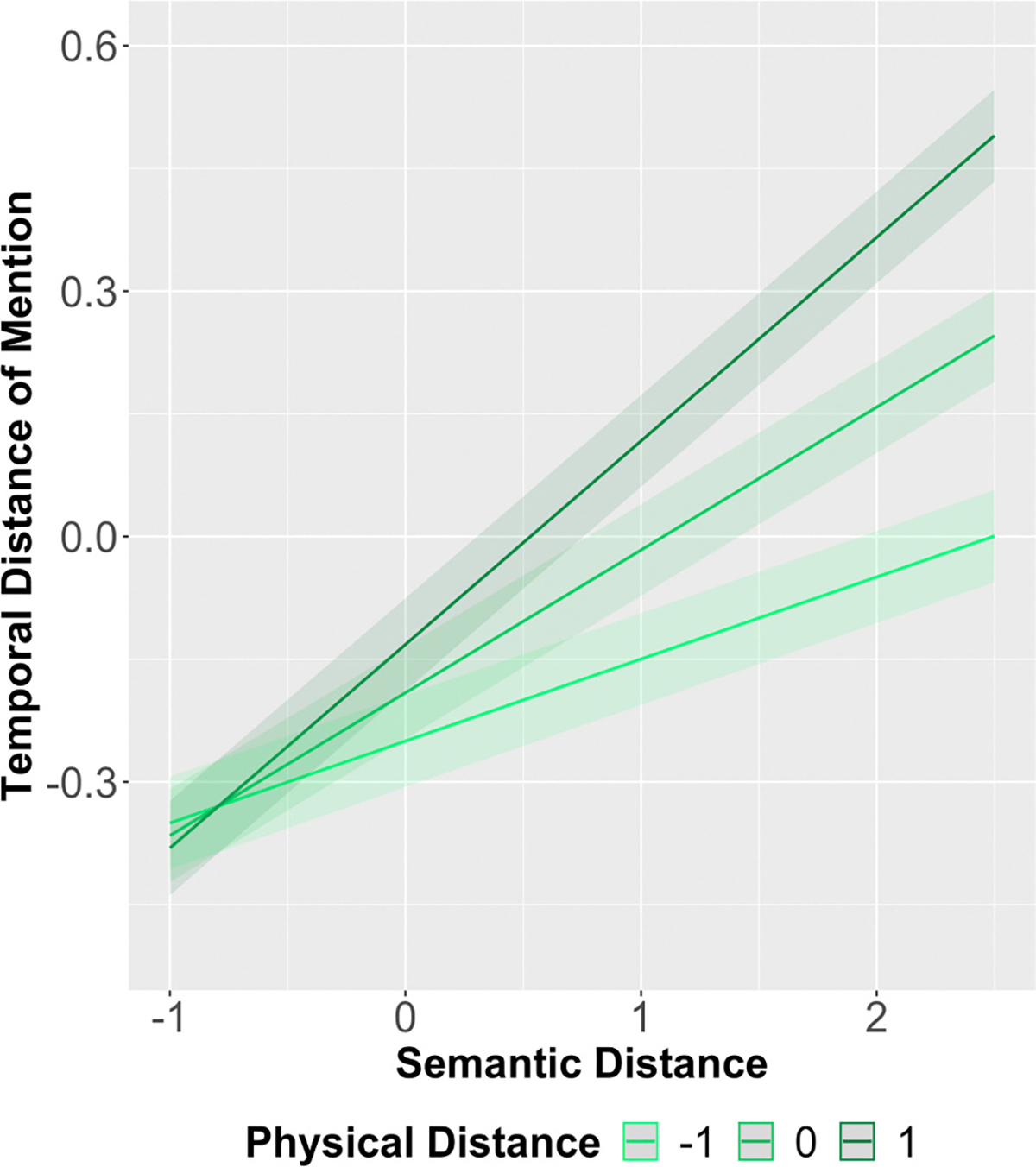
Model plots for the 2-way interaction between scaled physical distance and scaled semantic distance and the estimated scaled temporal distance of mention (y-axis). Model: Temporal distance ~ Semantic distance * Physical distance + (1|participant) + (1|scene) + (1|object_a) + (1|object_b). The plot shows that as semantic distance (x-axis) increases, temporal distance of mention (y-axis) also increases. It also shows that this effect interacts with physical distance. As physical distance increases (light to dark green lines), the temporal distance of mention also increases. Each line shows a different level of physical distance: 0 refers to the mean, −1 refers to −1 z score, and + 1 refers to +1 z score. Shaded regions indicate 95 % confidence intervals.

In support of the idea that descriptions have clusters, we found that objects that are mentioned close together tend to be close together in space and meaning (Fig. 4). We ran a linear mixed effects model with differences in onset times of mentioned objects as the outcome. We included physical distance and semantic distance as interacting fixed effects with random effects for subject, scene, and each of the objects. An initial model with random slopes failed to converge. The maximal converging model included a random intercept for subject, scene, and objects (random effect plots for subject and scene are available in the Supplemental Information 3). We preregistered a model that also included difference in center proximity and difference in size as interacting fixed effects, but these terms were highly correlated with physical distance. Because these effects are captured by physical distance and because we cannot interpret a mixed effects model with high collinearity, they were removed from the main analysis (collinearity analysis using Variance Inflation Factor and additional analyses for these features can be found in SI 4). The distance in onset, physical distance, and semantic distance were scaled around the grand mean using the R scale version 4.3.2 which standardizes the data. The model included weights for each pairwise relationship as discussed in the Methods section. We find a significant main effect of physical distance (*β* = 0.06, *t* = 25.80, *p* < 0.001) and semantic distance (*β* = 0.18, *t* = 86.57, *p* < 0.001). Objects that have shorter physical distance between them in the image and objects that are semantically more similar according to their labels are more likely to be discussed in close temporal proximity in verbal descriptions of the scene. The interaction term was also significant (*β* = 0.07, *t* = 53.47, *p* < 0.001), showing that the effect of physical distance increases with increased semantic distance. In other words, physical distance predicts temporal distance of object mentions more for objects that are semantically distant compared to objects that are semantically similar (Fig. 4). This suggests that when objects are highly semantically related, they are likely to be mentioned in close proximity in time regardless of physical distance, whereas semantically unrelated items are more likely to be mentioned soon after each other if they are closer together in space.

Next, we applied the same analysis to a randomized control dataset in an exploratory analysis. Due to the experiment’s design, which emphasized a naturalistic open-ended description, we could not create a condition in which participants did not organize their utterance. Given the novelty of the analysis procedure, we created a randomized control dataset for comparison where all the mentioned objects were identical to the collected dataset, but the order in which each object was mentioned was randomized. We assigned a random onset time chosen within the range of time in which participants were speaking for each description, where the minimum was the onset of the first word spoken and the maximum was the onset of the last word spoken. Onset times were randomly chosen with replacement, so multiple objects could have the same onset time as they did in the intact collected dataset. Verbal relations were then calculated using the same methodology as the intact dataset. We used the same scaling, coding, and model to analyze the randomized control dataset. In this dataset, we found no effect of physical distance (*β* = −0.0007, *t* = −0.87, *p* = −0.69) or semantic distance (*β* = −0.003, *t* = −0.39, *p* = 0.38) on the distance of mention, and we observed no interaction effect (*β* = 0.002, *t* = 1.14, *p* = 0.26).

### 3.3. Jumps between clusters

**Fig. 5.**
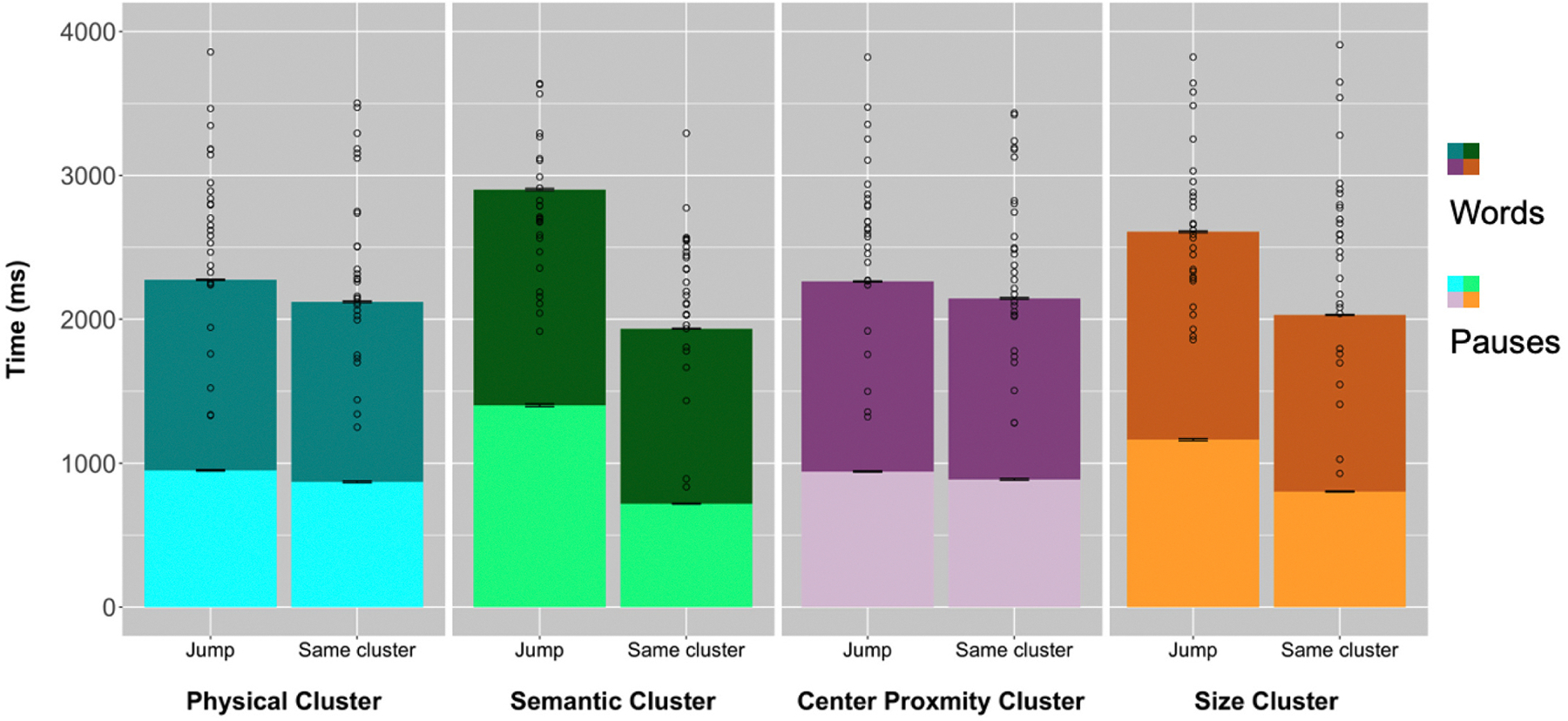
Each bar represents the mean total time between word onset times between subsequently mentioned objects for all participants. The bars have been split into speech time and silent pause time. The darker portion of the bar represents the sum of all word durations, and the light portion represents the sum of all silent pause durations. From the left, the faceted panel represents physical clusters, semantic clusters, center proximity clusters, and size clusters. The left bar for each faceted panel represents object transitions when the mentioned objects were in different clusters (jumps) and the right bar when they were in the same cluster. Error bars represent standard error. Circles represent the mean total time between word onset times between subsequently mentioned objects for each participant.

We next assessed whether the timing of the object labels provided in the descriptions was influenced by our predefined clusters. Because we were interested in the transition from one object in one cluster to the next object in another cluster and whether it differed from the transition between objects within a cluster, we looked only at the relationship between each object and the subsequent mentioned object. This analysis follows a similar approach from Hills et al. (2015), where they analyzed the time it took between naming one item and the next item. It is important to note that the clusters were defined by object properties identified during object segmentation before verbal descriptions were analyzed, and therefore could not be influenced by the descriptions. We found that people’s production of the next word takes substantially longer when jumping to an object that belongs to a new cluster compared to when they talk about another object within a cluster. This was true for all features (Fig. 5): physical similarity, semantic similarity, center proximity, and object size.

We ran a linear mixed effects model with differences in onset times of mentioned objects scaled over the grand mean, using the R scale which standardizes the data, as the outcome. Whether the next mentioned object was in the same cluster or a new cluster was coded as jumps (−1 for the same cluster and 1 for a new cluster). We included jumps in physical clusters, jumps in semantic clusters, jumps in size clusters, and jumps in center proximity clusters as interacting fixed effects. A collinearity analysis showed that the four features were not collinear (SI5; Marcoulides & Raykov, 2019). An initial model with random slopes failed to converge. The maximal converging model included a random intercept for subject, scene, and objects (random effect plots for subject and scene are available in the SI6). We found a significant main effect of jumps for all features (physical: *β* = 0.13, *t* = 20.72, *p* < 0.001; semantic: *β* = 0.10, *t* = 15.24, *p* < 0.001; center proximity: *β* = 0.07, *t* = 12.15, *p* < 0.001; size: *β* = 0.05, *t* = 7.40, *p* < 0.001). We also found interacting effects between physical and semantic jumps (*β* = 0.028, *t* = 4.575, *p* < 0.001), semantic and center proximity jumps (*β* = 0.020, *t* = 3.461, *p* < 0.001), semantic and size jumps (*β* = −0.023, *t* = −3.670, *p* < 0.001), and size and center proximity jumps (*β* = −0.015, *t* = −2.494, *p* = 0.013). Across all features, the direction of the effect was the same, such that jumping clusters took longer than staying in the same cluster. Therefore, the interactions indicated a difference in the magnitude of one effect given another. For objects across a physical jump, the effect of semantic jump on the temporal distance was higher compared to objects in the same physical cluster. This suggests that the transition between objects in different semantic clusters would take even longer if they were also in different physical clusters. For objects across a semantic jump, the effect of center proximity jump was higher and the effect of size jump was lower compared to objects in the same semantic cluster. For objects across a size jump, the effect of center proximity jump was lower compared to objects in the same size cluster. The full model output is provided in SI7.

### 3.4. Pauses and filler words

In an exploratory analysis, we investigated the use of silent pauses and filler words (e.g., “uh”, “um”) in participants’ descriptions. First, we calculated silent pause time by taking the total amount of time elapsed between word onset times of two subsequently mentioned objects and subtracting the word duration for every word uttered between them. This leaves us with the sum of all silent pauses between two subsequently mentioned objects. We ran the same model used to analyze jumps between clusters, with the pause time scaled over the grand mean as the outcome, jumps in physical clusters, jumps in semantic clusters, jumps in size clusters, and jumps in center proximity clusters as interacting fixed effects, and random effects for subject, scene, and each of the objects. We found a significant effect for each of the features (physical: *β* = 0.14, *t* = 20.94, *p* < 0.001; semantic: *β* = 0.10, *t* = 14.40, *p* < 0.001; center proximity: *β* = 0.07, *t* = 11.66, *p* < 0.001; size: *β* = 0.04, *t* = 6.32, *p* < 0.001) (Fig. 5) Interacting effects for pauses were similar to those of total temporal distance. (Refer to SI8 for full model).

**Fig. 6.**
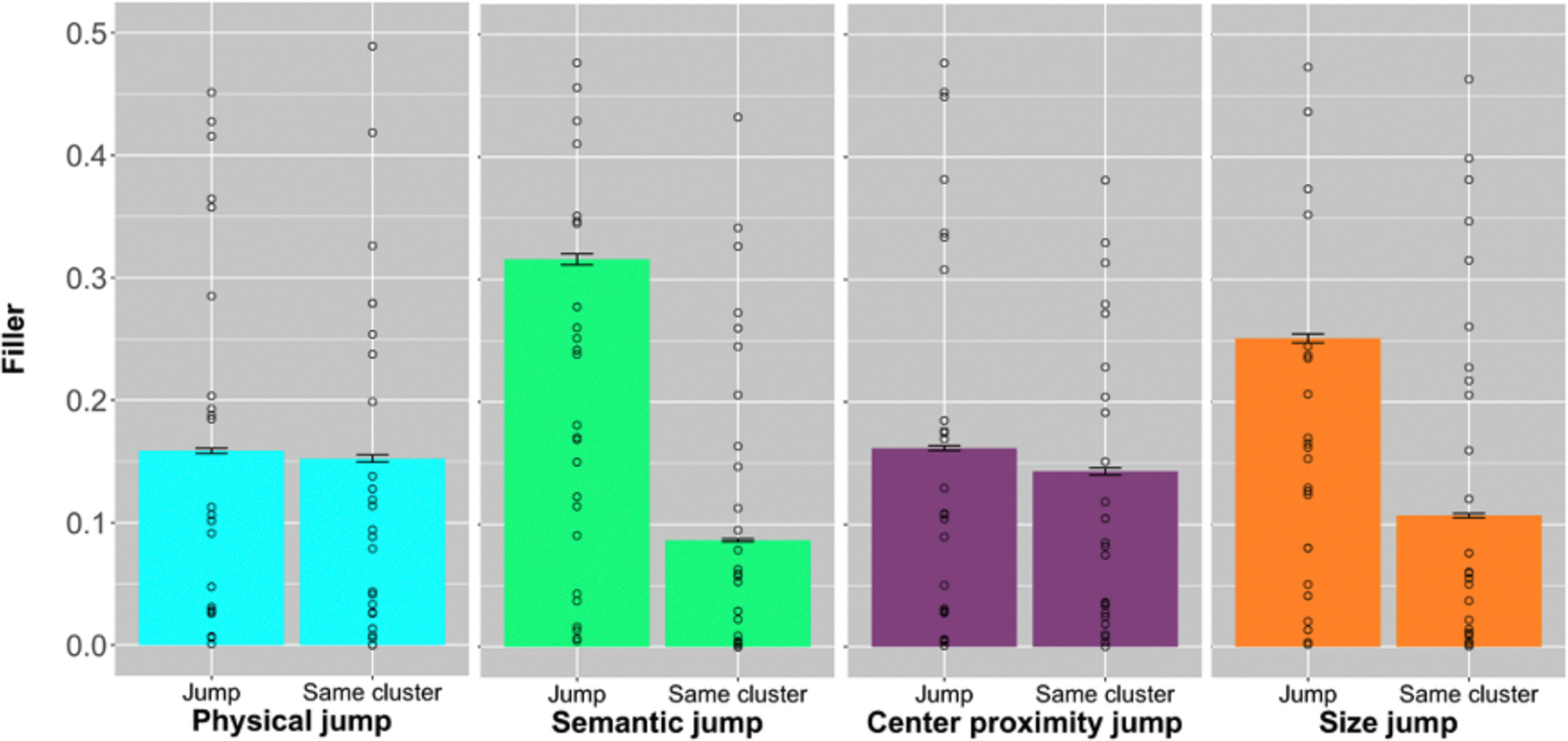
Each bar represents the mean number of fillers for all participants. From the left, the faceted panel represents physical clusters, semantic clusters, center proximity clusters, and size clusters. The left bar for each faceted panel represents object transitions when the mentioned objects were in different clusters (jumps) and the right bar when they were in the same cluster. Error bars represent standard error. Circles represent the mean total time between word onset times between subsequently mentioned objects for each participant.

Lastly, we analyzed the instances of filler words in the description, which included “uh”, “um”, and “umm” (i.e., lengthened “um”). We counted the number of times filler words appeared between two subsequently mentioned objects. We ran the same model used to analyze jumps between clusters, with the filler number as the outcome, jumps in physical clusters, jumps in semantic clusters, jumps in size clusters, and jumps in center proximity clusters as interacting fixed effects. We included random effects for subject, scene, and each of the objects. We found a significant effect for physical cluster, semantic cluster, and center proximity cluster, but not size cluster (physical: *β* = 0.03, *t* = 10.28, *p* < 0.001; semantic: *β* = 0.02, *t* = 9.28, *p* < 0.001; center proximity: *β* = 0.03, *t* = 10.76, *p* < 0.001; size: *β* = 0.003, *t* = 1.22, *p* = 0.22) (refer to SI 9 for full model). People were more likely to produce filler words between clusters compared to within clusters (Fig. 6). Visual inspection of Fig. 6 for size may appear as if there is a large difference in fillers for jumps and same clusters. However, this is likely because size is likely to be correlated with semantics (i.e., generally all chairs are the same size). An interacting model that includes both features, such as the one we used, is better suited to reveal effects of each feature. The exploratory analyses using pause time and filler words revealed that participants were more likely to be planning the next set of utterances when an object transition crossed a cluster boundary.

## 4. Discussion

How do we linearize complex ideas into a single sequence ready for speech? Linearization occurs at multiple levels from conception to articulation, with macroplanning being responsible for organization of communicative goals and subgoals (Levelt, 1989). Previous literature on linearization during microplanning investigated what is mentioned earlier or later in phrases and sentences and what it could tell us about production planning. In this paper, we took a different approach by asking how the relationships between ideas impacted the overall organization of the multiutterance production and the planning process on a larger time scale. By using scene descriptions, we were able to probe how relationships between objects in a scene affected the ordering and timing of object mentions in speech.

We found that the physical and semantic distances between objects predicted their temporal distance in the verbal descriptions. We also found that the time it takes from mentioning one object to the next object is significantly longer if the speaker jumps from one cluster to another compared to when they stay in the same cluster. Exploratory analyses looking at pause times and filler words suggest that this time is being used to plan the next sequence of utterance. Taken together, scene descriptions show evidence of object clustering and support the idea that these clusters facilitate strategic incremental production by allowing planning to be interleaved between clusters.

Our task tapped into several complex cognitive problems that benefited from the creation of clusters. First, scene description is a linearization problem. Given the complex real-world images with many objects that our participants were required to describe, participants needed to choose what to talk about and how. Part of the linearization problem is that the resulting utterance should follow a pattern where, more often than not, the last thing that is mentioned is related to the next thing to be discussed (Levelt, 1989). We found that objects that are close together in physical and semantic distance are mentioned close together in descriptions, resulting in a coherent sequence of objects.

To organize the utterance, one must also tackle the planning problem – when do you plan the order of objects and how much do you plan? Clustering facilitated this process by enabling an incremental planning approach. A participant would first group objects into clusters, as a type of preliminary plan. They can begin their production of the first cluster, describing objects within that cluster. They can then plan their next set of utterances while moving to the next cluster. Consistent with this proposed strategy, we found that the time it takes to transition from one object to another is significantly longer if the speaker jumps from one cluster to another compared to when they stay in the same cluster. Exploratory analyses also showed that pause times and fillers were more evident at these cluster boundaries. Given prior work showing that pauses and filler words are linguistic signals of planning, it suggests that this time is being used to plan the next sequence of utterance (Clark, 2002; Ferreira, 1991, 1993).

Incremental planning through clustering combines advantages of planning and incrementality. On one hand, planning at the cluster level, as opposed to the object-level, reduces the number of choices the speaker needs to make, allowing them to start speaking after making only a preliminary plan. A complete plan, on the other hand, may take several minutes instead. This strategy also allows for planning to continue after speech initiation at designated times, such as at cluster boundaries, instead of between every message. Within clusters, incrementality can take the wheel. Here, we do not mean to imply that planning can *only* take place between clusters and incrementality can *only* take place within clusters, but rather that there is a relative shift in how much we are planning and how much we are being incremental in relation to cluster boundaries.

While our task examined macroplanning and differed from other production tasks designed to examine microplanning, our results are not incompatible with previous literature on linearization. Factors related to cognitive accessibility such as agency, animacy, word-length, and frequency are known to influence what is mentioned earlier in production (Christianson & Ferreira, 2005; Griffin & Bock, 2000; MacDonald, 2013; Morgan & Levy, 2016; Tachihara & Goldberg, 2020). In our paradigm, this pattern may translate to more frequent words produced earlier within a cluster and less frequent words later within the cluster. Barker et al., 2023 used the same task as the current study and investigated the overall order of mention of the objects throughout the entire description. Surprisingly, they found that frequency of words did not predict the order of mention. If we consider only a flat sequence, this finding may run counter to the well-established frequency effect in production. However, if we consider a clustering strategy where incrementality is reserved for choices within a cluster, it may help explain why no effect was found in the overall order of mention.

Another factor that has been identified in the production ordering literature is animacy and agency. A limitation of this study is the use of scenes which are made up of purely inanimate items. However, we chose to use scenes without animate entities because animate entities are strongly attention-capturing (Clarke et al., 2013). By restricting the stimuli to inanimate objects, we were able to look at organization of objects without an interaction of animacy. The open-ended nature of the task allows future work to examine how humans or other animate entities and their actions within a scene may change how speakers cluster and organize their descriptions. A controlled set of visual display created by the experimenter that systematically manipulates objects, their properties in previously identified factors influencing production, and their semantic and physical relationships may also reveal further details about how these various factors interact and influence clustering in descriptions. A controlled set of stimuli may also be able to compare different types of semantic relationships. For example, shared category membership (ex: fork and knife) and associative links (ex: knife and steak) are known to change the ease of production (Abdel Rahman & Melinger, 2009; Cutting & Ferreira, 1999). As we begin to understand multiutterance descriptions, the natural scenes used in the current experiment were useful because it allowed us to tap into spontaneous organization of scene descriptions in an ecologically valid manner using a data-driven approach to clustering. However, future research that manipulates the scene is needed to disentangle factors related to clustering at a finer scale.

Future research manipulating and examining the relationship between words, concepts, and clusters may also be able to shed light on the similarity, differences, and interactions between macroplanning and microplanning. Do the same factors that influence microplanning influence macroplanning? Do the choices made in one stage impact the other? Levelt (1989) discusses the possibility that macroplanning and microplanning may impact one another bidirectionally (see also Garrett, 1989). While no doubt the choice of which concept to talk about will affect downstream choices such as lexical retrieval, it is also possible that the specific word choice will affect the choice of the next concept. For example, choosing the word “stool” instead of “chair” or “furniture” may lead the speaker to talk about “a bar” next. It remains an open question whether macroplanning and microplanning are two distinct stages or whether they interact. Future work should try to investigate how the linearization process changes with the scope of the production. We believe the use of clusters may be helpful in examining this question.

In this paper, we examined four features of interest: physical distance, semantic distance, center proximity, and size. The choice of these features was motivated by previously identified features relevant in scene viewing as well as the hypothesis that clusters would be related to the time and effort it takes to retrieve the next item. Why is clustering by physical and semantic distance beneficial during this task? First, physical distance of the objects in the image is closely related to the saccade amplitude or the distance that the eye travels from one location to another. Longer eye movements take more time to program and are more error prone. In general, there is a strong bias for viewers to make shorter saccades in scenes (Henderson, 2020). Physical distance is also related to visual acuity, with objects farther in the periphery more difficult to see and identify. Similarly, shorter semantic distance is also associated with less effort in a production task. The activation of one item spreads through associative links and primes semantically related items, making the retrieval and production of the subsequent item easier (Hills et al., 2015).

A strategy to cluster by physical and semantic distance may be beneficial especially when trying to reduce the burden on working memory during multiutterance production. Remember that on a radical planning view, the order of items must be remembered precisely so that it can be produced one at a time. We know that clustering is useful in memory (Miller, 1956; Thalmann et al., 2019). Physical and semantic clusters are particularly useful because they do not need to be kept in working memory. Because participants have the scene in front of them as they are describing it, they can simply rely on the visible properties in the image, like physical distance, rather than remembering a random order. Semantic clustering is also helpful because it relies on long-term knowledge. While these scenes are presumably novel to each participant, by using naturalistic images that contained familiar scenes and objects, participants were likely able to use their rich associative network to help cluster the objects in the scene.

Semantic relationships have been extensively tested in production tasks, sometimes leading to a facilitation effect via semantic priming and sometimes an interference effect via competition of semantic neighbors (Abdel Rahman & Melinger, 2009, 2019; Cutting & Ferreira, 1999; Rabovsky et al., 2021). Why did this task show results that are consistent with facilitation effects rather than interference effects? While semantic priming is always present, interference effects are observed only when the competition effect outweighs the initial activation from semantic priming. Therefore, interference effects are observed less often, are more prone to contextual effects (Abdel Rahman & Melinger, 2009) and may be overestimated in the literature (Lampe et al., 2022). Notably, interference effects are commonly observed in paradigms where there is a target stimulus and a second distractor that is related to the target. The related distractor must be suppressed when retrieving the word for the target, making production less fluid. This differs from our task in that in a scene description task, participants have the choice in what to name and are not given a target.

Support for the idea that the lack of choice leads to an interruption in production comes from code switching experiments in bilingual populations. In Blanco-Elorrieta and Pylkkänen (2017), participants completed a picture naming task in various language switching contexts. They found that when the choice of output language was voluntary, there was no switching cost, compared to when the output language was experimentally determined with a colored box cue. Competition between languages only led to longer speech initiation times when there was no choice and participants had to produce a target answer. While Blanco-Elorrieta and Pylkkänen (2017) is about cross-language competition, similar effects of target and task goal is likely to explain semantic competition effects found in picture naming tasks. Given the flexibility in our task, semantic priming is more likely to have a stronger effect than any interference effects from semantically related items. Additionally, semantic effects may differ between macroplanning and microplanning stages. The semantic interference effects reviewed above occur when words are retrieved during microplanning. It is unknown whether macroplanning also exhibits semantic interference effects. In either case, we did not observe it in our task.

We acknowledge that the advantage that comes from utilizing physical and semantic distances we have discussed so far is closely-related to the task and its goals. It is more than likely that a description of a scene without any animate object will inherently lead to descriptions that are about the spatial relationships of the objects. Previous work has shown that the task-dependent goals, such as describing the scene versus describing what a person would do in a scene, can alter which features impact where people attend to in a scene (Rehrig et al., 2022). The interaction effect we found, in which physical distance of objects was a predictor of the temporal distance of mention especially as semantic distance increased, is likely a result of an image-based task and may differ for other types of multiutterance production situations. We invite future work to examine other features as well as how task-specific goals might impact the strength of these features and their interactions.

Language production may be viewed as a type of decision-making task, because the speaker is continuously deciding the content and the form of the utterance (Ferreira et al., 2009; Levelt, 1989; MacDonald, 2013). Uncertainty during this decision-making process can even impact word order during production (Gussow & MacDonald, 2023). In this scene description task, participants needed to find objects to describe. Therefore, this language production task can also be viewed as a foraging problem, a type of decision-making process. In a traditional foraging task, an animal tries to get as much food as possible given a patchy environment. Animals do so by balancing local exploitation (i.e., staying on the same tree to find more apples) and global exploration (i.e., traveling to a new tree to find more apples). Similarly, during descriptions, people need to decide whether to keep talking about a specific area of the scene or move on to a new area. Foraging can be externally driven, like moving from one object to the next based on their physical distance in the image, but it can also be internally driven, like choosing to describe an object that is semantically related to the last-mentioned object.

The advantage of exploitation, for animals foraging for food and for scene description, is minimized effort and reduced time to get to the next target (i.e., apples for animals and next object for descriptions). The least effortful strategy may seem like it would be to keep choosing physical or semantically near items. However, staying in the same cluster for too long or “overharvesting,” may not be appropriate given the context (Harhen & Bornstein, 2023). The disadvantages of exploitation include increased effort and diminished return at a given cluster. For example, if the speaker is talking about a semantic cluster of appliances, there may not be more appliances or easily namable appliances in that scene. Word frequency and other factors related to ease of production may serve as a signal to the speaker that they should leave the cluster. In general, staying in the same area would lead to a description that is overly specific and fails to describe the rest of the scene. Given that the task did not specify how to describe the scene, any type of description, specific or general, would suffice. Participants’ tendency to jump clusters despite its added effort hints at their goals during the task. The guiding principle of descriptions may be a balance between exploitation and exploration, which is enabled by the clustering of objects. This balance is a characteristic feature of foraging behavior.

In conclusion, scene descriptions show evidence of object clustering and support the idea that clustering facilitates strategic incremental planning during multiutterance production. This clustering in turn promotes speakers’ adoption of an explore-exploit balancing strategy for accomplishing their linguistic task. Overall, the findings support a view of language production that treats the generation of complex descriptions as a type of foraging task, with episodes of exploitation alternating with periods of exploration.

## Supplementary Material

Planning to be incremental: supplementary items

## Data Availability

Data and code are available on OSF View only. Link in included on manuscript.

## Appendix A. Supplementary data

Supplementary data to this article can be found online at https://doi.org/10.1016/j.cognition.2025.106330.
