# Supplementary material for: Planning to be incremental: Scene descriptions reveal meaningful clustering in language production
Source: Cognition. Author manuscript; Available in PMC 2026 Mar 17. (PMC12994004; doi:10.1016/j.cognition.2025.106330)
Supplement: Planning to be incremental: supplementary items [file NIHMS2149096-supplement-Planning_to_be_incremental__supplementary_items.docx]

**Supplemental Information**

SI 1. Guidelines for segmentation.

We followed the following guidelines when segmenting objects in a scene.

· Each object was segmented separately (ex: one polygon for one bottle & another polygon for another bottle). However, in some cases the Segment Anything Model (SAM) was not able to separate each object because the objects are too small or visually hard to distinguish. In such cases, one polygon was created with multiple objects (ex: one polygon for 6 glasses).

· Objects were segmented as a whole. If SAM was able to identify parts of objects, the parts were also segmented in addition to the whole object segmentation (ex: a side table with a drawer resulted in 2 polygons – one for the whole side table and one for the drawer).

· Every effort was made to name every object in the image. However, objects that could not be identified were removed from analysis.

· Overlaps between polygons were avoided except when;

o A polygon for the part of an object overlapped with the whole object.

o Objects were see-through (ex: buildings seen through a window).

o When the objects could not be segmented separately because they were too small.

SI 2. Guidelines for verbal data coding.

We followed the following guidelines when identifying objects in the transcription.

· For rephrases, repairs, or homing, we chose the latest word used (ex: for “chair I mean stools”, stool was identified as the word referring to the object).

· “Each” was interpreted the same as “all”. If there were 6 chairs and the transcript indicated “each chair,” all 6 chairs were associated with “chair.”

· General words were associated with all objects in that category. For “furniture,” we chose all the furniture in the scene.

· Pronouns were also identified as an object referent. (ex: “I see a chair. It is gray.” “It” was also identified as an object referent to the chair.) Pronouns included it, they, them, those, that, one, other, and more.

· For compound words, the head noun was used. For “speaker system”, “system” was the word associated with the object.

· In conjunctions, both objects were identified. For example, for “I see bottles and glasses,” bottles were associated with bottles and glasses are associated with glasses.

SI 3. Random effect plots for distance model.

Temporal distance ~ Physical distance * Semantic distance + (1|participant) + (1|scene)+ (1|object_a)+ (1|object_b)


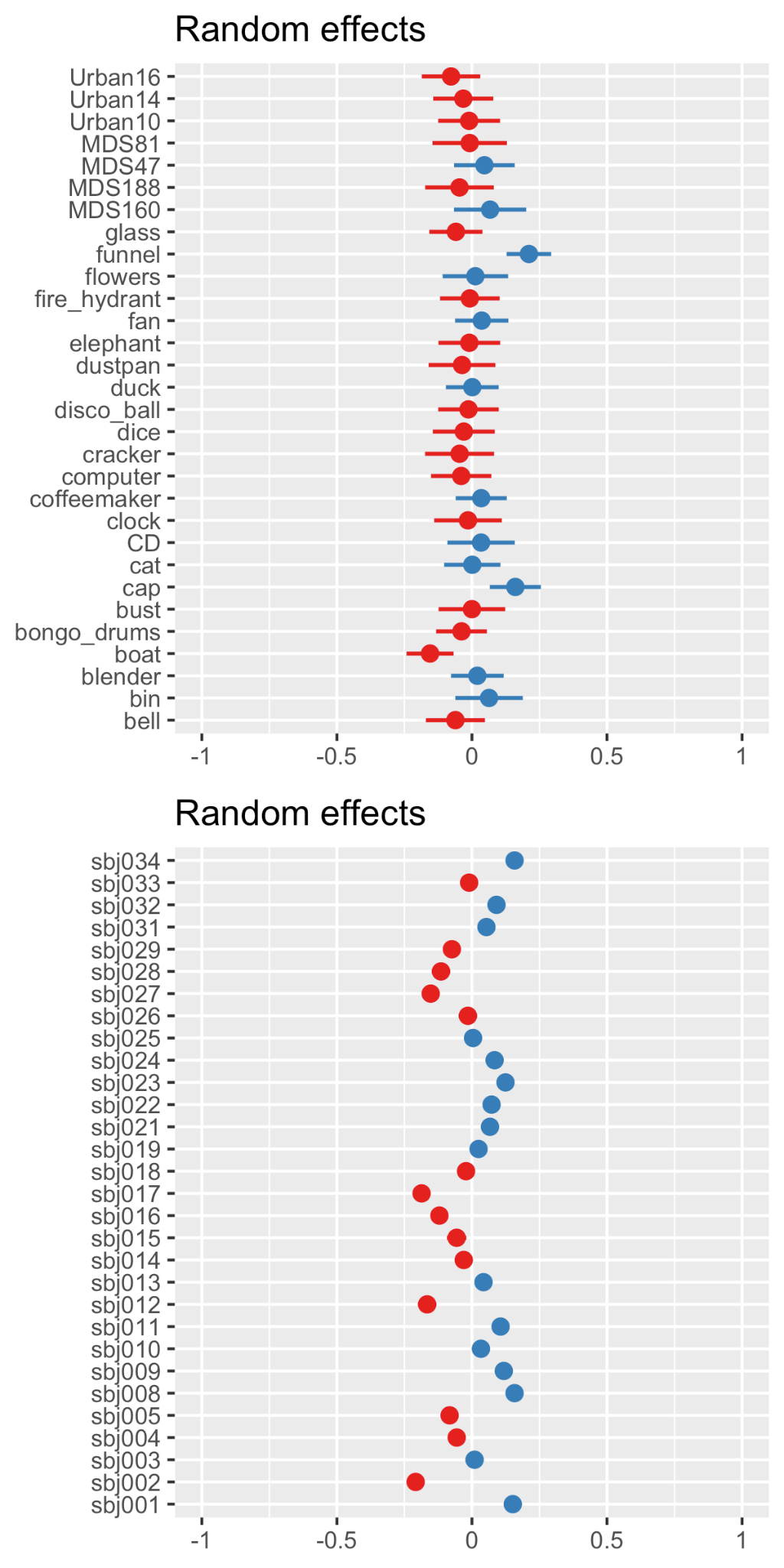


SI 4. Effects of difference in center proximity and difference in size.

Result of the collinearity analysis for the full model with physical distance, semantic distance, difference in size and difference in center proximity as interacting fixed effects. Variance Inflation Factor (VIF) measures how much the variance of a coefficient is inflated due to linear dependencies with other predictors. A VIF of 1 indicates no correlation, while values above 5 typically signal problematic multicollinearity.

| **Term** | **VIF** | **VIF_CI_low** | **VIF_CI_high** |
| --- | --- | --- | --- |
| dist_phys_scaled | 8.32 | 8.28 | 8.37 |
| dist_sem_scaled | 1.96 | 1.95 | 1.97 |
| diff_center_scaled | 6.36 | 6.33 | 6.39 |
| diff_size_scaled | 11.44 | 11.38 | 11.50 |
| dist_phys_scaled:dist_sem_scaled | 7.56 | 7.52 | 7.60 |
| dist_phys_scaled:diff_center_scaled | 6.94 | 6.90 | 6.97 |
| dist_sem_scaled:diff_center_scaled | 6.26 | 6.23 | 6.29 |
| dist_phys_scaled:diff_size_scaled | 23.32 | 23.19 | 23.44 |
| dist_sem_scaled:diff_size_scaled | 10.82 | 10.77 | 10.88 |
| diff_center_scaled:diff_size_scaled | 22.44 | 22.32 | 22.56 |
| dist_phys_scaled:dist_sem_scaled:diff_center_scaled | 6.55 | 6.51 | 6.58 |
| dist_phys_scaled:dist_sem_scaled:diff_size_scaled | 23.03 | 22.91 | 23.16 |
| dist_phys_scaled:diff_center_scaled:diff_size_scaled | 23.35 | 23.22 | 23.47 |
| dist_sem_scaled:diff_center_scaled:diff_size_scaled | 23.09 | 22.97 | 23.22 |
| dist_phys_scaled:dist_sem_scaled:diff_center_scaled:diff_size_scaled | 23.93 | 23.81 | 24.06 |

Result of the collinearity analysis for the limited model with physical distance and semantic distance as interacting fixed effects.

| **Term** | **VIF** | **VIF CI low** | **VIF CI high** |
| --- | --- | --- | --- |
| dist_sem_scaled | 1.26 | 1.26 | 1.26 |
| dist_phys_scaled | 3.41 | 3.4 | 3.43 |
| dist_sem_scaled:dist_phys_scaled | 3.14 | 3.12 | 3.15 |

Given that the difference in center proximity and difference in size could not be included in the full model, we analyzed each effect in separate models with one fixed effect. We found that difference in center proximity and difference in size were each significant predictors of difference in onset times for mentioned objects (center proximity *β =* 0.11*, t* = 83.99, *p* < 0.001; size *β =* 0.02*, t* = 13.53, *p* < 0.001, respectfully).

SI 5. Collinearity analysis for jumps.

| **Term** | **VIF** | **VIF CI low** | **VIF CI high** |
| --- | --- | --- | --- |
| Physical jump | 1.58 | 1.56 | 1.59 |
| Semantic jump | 1.51 | 1.49 | 1.52 |
| Size jump | 1.47 | 1.46 | 1.49 |
| Center proximity jump | 1.45 | 1.44 | 1.47 |
| Physical jump:Semantic jump | 1.57 | 1.55 | 1.59 |
| Physical jump:Size jump | 1.56 | 1.55 | 1.58 |
| Semantic jump:Size jump | 1.54 | 1.53 | 1.56 |
| Physical jump:Center proximity jump | 1.43 | 1.42 | 1.45 |
| Semantic jump:Center proximity jump | 1.55 | 1.54 | 1.57 |
| Size jump:Center proximity jump | 1.47 | 1.46 | 1.49 |
| Physical jump:Semantic jump:Size jump | 1.60 | 1.59 | 1.62 |
| Physical jump:Semantic jump:Center proximity jump | 1.58 | 1.57 | 1.60 |
| Physical jump:Size jump:Center proximity jump | 1.61 | 1.60 | 1.63 |
| Semantic jump:Size jump:Center proximity jump | 1.60 | 1.58 | 1.61 |
| Physical jump:Semantic jump:Size jump:Center proximity jump | 1.60 | 1.59 | 1.62 |

SI 6. Random effect plots for jump model.

Difference in onset ~ Physical jump * Semantic jump * Size jump * Center proximity jump + (1|participant) + (1|scene)+ (1|object_a)+ (1|object_b)


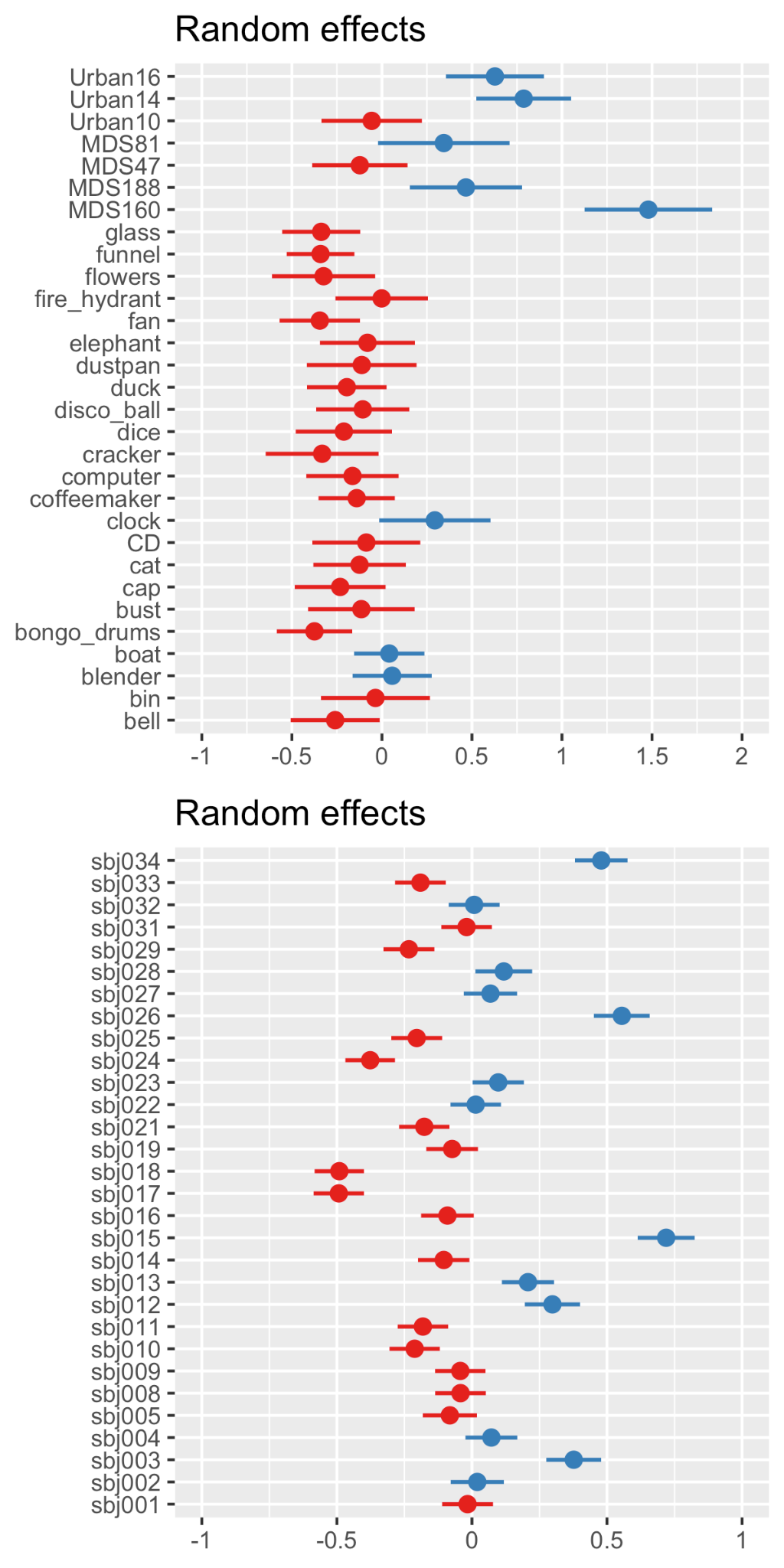


SI 7. Model output for jump analysis

Estimate refers to the beta values. * refers to p <0.05. ** refers to p<0.01. *** refers to p<0.001.

Temporal distance ~ Physical jump * Semantic jump * Size jump * Center proximity jump + (1|participant) + (1|scene)+ (1|object_a)+ (1|object_b)

| **Fixed Effects** | **Estimate** | **t value** | **p value** | **Column1** |
| --- | --- | --- | --- | --- |
| (Intercept) | 0.307 | 3.192 | 0.003 | ** |
| jump_phys | 0.130 | 20.717 | <0.001 | *** |
| jump_sem | 0.097 | 15.235 | <0.001 | *** |
| jump_size | 0.048 | 7.396 | <0.001 | *** |
| jump_center | 0.073 | 12.148 | <0.001 | *** |
| jump_phys:jump_sem | 0.028 | 4.575 | <0.001 | *** |
| jump_phys:jump_size | -0.008 | -1.357 | 0.175 |  |
| jump_sem:jump_size | -0.023 | -3.670 | <0.001 | *** |
| jump_phys:jump_center | -0.005 | -0.818 | 0.414 |  |
| jump_sem:jump_center | 0.020 | 3.461 | <0.001 | *** |
| jump_size:jump_center | -0.015 | -2.494 | 0.013 | * |
| jump_phys:jump_sem:jump_size | 0.022 | 3.719 | <0.001 | *** |
| jump_phys:jump_sem:jump_center | -0.038 | -6.352 | <0.001 | *** |
| jump_phys:jump_size:jump_center | 0.019 | 3.204 | 0.001 | ** |
| jump_sem:jump_size:jump_center | 0.026 | 4.328 | <0.001 | *** |
| jump_phys:jump_sem:jump_size:jump_center | -0.016 | -2.671 | 0.008 | ** |

SI 8. Model output for pause time

Pause time ~ Physical jump * Semantic jump * Size jump * Center proximity jump + (1|participant) + (1|scene)+ (1|object_a)+ (1|object_b)

| **Fixed effects** | **Estimate** | **t value** | **p value** |  |
| --- | --- | --- | --- | --- |
| (Intercept) | 0.372 | 4.074 | <0.001 | *** |
| Physical jump | 0.135 | 20.936 | <0.001 | *** |
| Semantic jump | 0.095 | 14.395 | <0.001 | *** |
| Size jump | 0.042 | 6.320 | <0.001 | *** |
| Center proximity jump | 0.072 | 11.656 | <0.001 | *** |
| Physical jump:Semantic jump | 0.026 | 4.102 | <0.001 | *** |
| Physical jump:Size jump | -0.010 | -1.584 | 0.113 |  |
| Semantic jump:Size jump | -0.029 | -4.490 | <0.001 | *** |
| Physical jump:Center proximity jump | 0.002 | 0.295 | 0.768 |  |
| Semantic jump:Center proximity jump | 0.013 | 2.188 | 0.029 | * |
| Size jump:Center proximity jump | -0.014 | -2.271 | 0.023 | * |
| Physical jump:Semantic jump:Size jump | 0.021 | 3.352 | 0.001 | *** |
| Physical jump:Semantic jump:Center proximity jump | -0.028 | -4.625 | <0.001 | *** |
| Physical jump:Size jump:Center proximity jump | 0.011 | 1.821 | 0.069 | . |
| Semantic jump:Size jump:Center proximity jump | 0.026 | 4.185 | <0.001 | *** |
| Physical jump:Semantic jump:Size jump:Center proximity jump | -0.014 | -2.265 | 0.023 | * |

SI 9.

Filler ~ Physical jump * Semantic jump * Size jump * Center proximity jump + (1|participant) + (1|scene)+ (1|object_a)+ (1|object_b)

| **Fixed effects** | **Estimate** | **t value** | **p value** |  |
| --- | --- | --- | --- | --- |
| (Intercept) | 0.245 | 5.986 | <0.001 | *** |
| Physical jump | 0.027 | 10.228 | <0.001 | *** |
| Semantic jump | 0.024 | 9.281 | <0.001 | *** |
| Size jump | 0.003 | 1.217 | 0.224 |  |
| Center proximity jump | 0.027 | 10.759 | <0.001 | *** |
| Physical jump:Semantic jump | 0.008 | 3.300 | 0.001 | *** |
| Physical jump:Size jump | 0.000 | -0.006 | 0.995 |  |
| Semantic jump:Size jump | 0.003 | 1.083 | 0.279 |  |
| Physical jump:Center proximity jump | 0.006 | 2.402 | 0.016 | * |
| Semantic jump:Center proximity jump | 0.006 | 2.357 | 0.018 | * |
| Size jump:Center proximity jump | -0.012 | -5.053 | <0.001 | *** |
| Physical jump:Semantic jump:Size jump | 0.006 | 2.481 | 0.013 | * |
| Physical jump:Semantic jump:Center proximity jump | 0.001 | 0.252 | 0.801 |  |
| Physical jump:Size jump:Center proximity jump | 0.000 | 0.002 | 0.999 |  |
| Semantic jump:Size jump:Center proximity jump | 0.004 | 1.489 | 0.136 |  |
| Physical jump:Semantic jump:Size jump:Center proximity jump | -0.008 | -3.198 | 0.001 | ** |
